# Case Report: Presumptive spinal embryonal tumor in a cat

**DOI:** 10.3389/fvets.2025.1633279

**Published:** 2025-09-19

**Authors:** Marieke van den Heuvel, Quinten Van Koulil, Dorien Willems, Ines Carrera, Wilhelmina Bergmann, Daniel R. Rissi, Koen M. Santifort

**Affiliations:** ^1^IVC Evidensia Small Animal Referral Hospital Hart Van Brabant, Neurology, Waalwijk, Netherlands; ^2^Vet Oracle Teleradiology, Norfolk, United Kingdom; ^3^Division of Pathology, Department of Biomolecular Health Sciences, Veterinary Pathology Diagnostic Centre, Faculty of Veterinary Medicine, Utrecht University, Utrecht, Netherlands; ^4^Athens Veterinary Diagnostic Laboratory, Department of Pathology, College of Veterinary Medicine, University of Georgia, Athens, GA, United States; ^5^IVC Evidensia Small Animal Referral Hospital Arnhem, Neurology, Arnhem, Netherlands

**Keywords:** primitive neuroectodermal tumor, cauda equina, conus medullaris, surgery, histology

## Abstract

A 5-year-old male castrated domestic shorthaired cat was presented with asymmetrical, right worse than left, progressive paraparesis, proprioceptive ataxia, and a flaccid tail. Neuroanatomical localization was to the L4-caudal spinal cord segments, nerve roots, and/or spinal nerves worse on the right side. Based on development of clinical signs, the spinal cord segments and/or spinal nerves forming the cauda equina were suspected to have been involved first. Magnetic resonance imaging (MRI) revealed a well-defined lesion extending from the level of the mid-body of L5 to the caudal aspect of L6, slightly heterogeneous, hyperintense in T2W and STIR, mildly hypointense in T1W, and with mild to moderate contrast enhancement. Initial prednisolone treatment (0.6 mg/kg twice daily) failed to result in improvement, and the neurological status worsened. Surgery (L5-L6 dorsal laminectomy) revealed a red, intradural and predominantly extraparenchymal mass that was partly fused with the conus medullaris and spinal nerve roots. Euthanasia was elected intraoperatively after discussion with the owner. Histologically the mass consisted of a poorly demarcated primarily extraparenchymal but also intraparenchymal neoplasm composed of tall, columnar neoplastic cells arranged in rows and pseudostratified patterns aligned perpendicularly to the fibrovascular stroma. Neoplastic cells formed neuroblastic rosettes and pseudorosettes throughout. The mitotic count was 73 in 2.37 mm^2^ (10 FN22/40X fields). Neoplastic cells had widespread cytoplasmic immunolabeling for PGP9.5 and were negative for pancytokeratin, OLIG2, GFAP, NeuN, NSE, NF, and SYN. Histologic changes were consistent with a presumed embryonal tumor.

## Introduction

Primary spinal cord neoplasms are infrequently reported in cats, accounting for approximately 16% of all feline spinal cord and vertebral neoplasms (1). The predominant primary neoplasms of the spinal cord are meningiomas and gliomas, accounting for approximately 7 and 8%, respectively, of all tumors involving the spinal cord and vertebrae (1). Meningiomas, arising from arachnoid (cap) cells, are slow-growing tumors and affect mainly adult to older cats (1). Among gliomas, astrocytomas are the most frequent, followed by oligodendrogliomas and ependymomas (2). These tumors primarily affect adult to aged cats, without sex or breed predisposition. Spinal gliomas in cats so far reported, have primarily affected the cervical spinal cord, but cases involving the thoracic, lumbar, and sacrocaudal segments have been reported as well (2–12).

Embryonal central nervous system (CNS) neoplasms are rare and arise from undifferentiated or germinal primitive neuroepithelial cells capable of multi-lineage differentiation, including neuronal, glial, and ependymal lineages. Only five cases of intraparenchymal primary embryonal CNS neoplasms have been reported in cats (13–17). Olfactory neuroblastomas originating from the olfactory neuroepithelium are more frequently documented and occur in the caudal nasal cavity and olfactory bulbs (18).

In humans, the classification of an embryonal CNS neoplasm is based on its molecular features, and most tumors are categorized as medulloblastomas (19). In domestic animals, the diagnosis of embryonal neoplasms is still dependent on histology and immunohistochemistry (IHC), but not enough cases have been reported to determine their specific diagnostic features (20). While infratentorial neoplasms arising from the cerebellum or pons are typically classified as medulloblastomas, embryonal neoplasms arising from other areas in the CNS may be categorized as medulloepithelioma, neuroblastoma, ganglioneuroblastoma, or simply as embryonal neoplasm (20). Previous (human and veterinary) literature often referred to such neoplasms as ‘primitive neuroectodermal tumors (PNETs)’ but this term is no longer considered appropriate (20–22).

In this case report, we describe a presumed embryonal tumor affecting the lumbar and sacrocaudal spinal cord and adjacent spinal nerves of the cauda equina in a cat.

## Case description

A 5-year-old castrated male European Shorthair cat was presented to the emergency clinic with a subacute onset of progressive tail flaccidity accompanied by difficulty walking and jumping. The owner did not report fecal or urinary incontinence at that time. General examination revealed no abnormalities. Upon neurological evaluation, the cat showed a normal mental status and behavior. The cat had a bilateral plantigrade stance, ambulatory paraparesis, and proprioceptive ataxia, worse on the right side, and a flaccid tail (Supplementary Video 1). Proprioceptive deficits were observed in both pelvic limbs, more pronounced on the right side. Spinal reflex testing showed reduced withdrawal reflexes in both pelvic limbs and absent perineal reflex. Bilateral atrophy of the pelvic limb muscles was noted. The anal sphincter tone was decreased. Cranial reflexes and responses were normal. Hyperesthesia was observed in the lumbosacral region. Nociception was intact in all four limbs and dubious in the tail.

**Figure 1:**
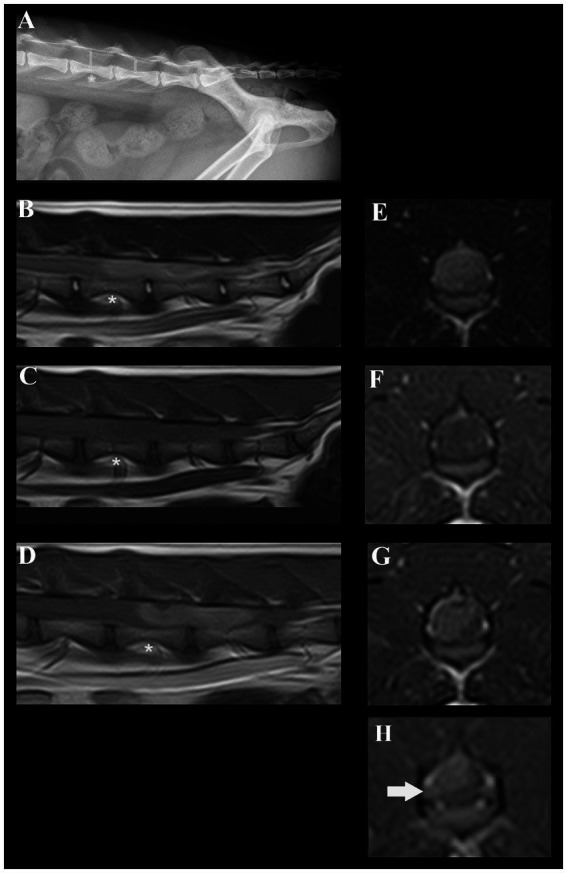
Radiographic and magnetic resonance images of a cat diagnosed with a presumed embryonal tumor affecting the spinal cord. **(A)** Lateral radiograph of the vertebral column. The dorsoventral spinal canal height is subjectively increased centered at the level of the L5 and L6 vertebrae (red lines, red asterisk at the level of L5). **(B)** Sagittal plane T2W MRI; a slightly heterogeneous, mainly hyperintense space occupying lesion is present in the spinal canal. Red asterisk at the level of L5. **(C)** Sagittal plane T1W MRI; a mildly hypointense space occupying lesion is present in the spinal canal. Red asterisk at the level of L5. **(D)** Sagittal plane T1W + contrast; moderately heterogeneous contrast-enhancement of the lesion can be noted. Red asterisk at the level of L5. **(E)** Transverse plane T2W at the level of L5; the moderately well-defined right sided mass lesion can be identified. **(F)** Transverse plane T1W at the level of L5; the lesion is mildly hypointense. **(G)** Transverse plane T1W + contrast at the level of L5; moderately heterogenous enhancement of the lesion can be identified. **(H)** Contrast-enhancement and mild swelling of the right-sided L6 nerve root can be appreciated.

Neuroanatomical localization was to the L4-caudal spinal cord segments, nerve roots, and/or spinal nerves worse on the right side. Our differential diagnoses included predominantly degenerative (intervertebral disc degeneration and herniation), anomalous, neoplastic, and inflammatory processes. Lateral radiographs (acquired by the referring veterinarian) revealed a subjectively increased dorsoventral height of the spinal canal centered at the level of the L5 and L6 vertebrae (Figure 1A). The cat was anesthetized and positioned in dorsal recumbency for a magnetic resonance imaging (MRI) study of the thoracolumbar spinal cord (1.5 T Canon Vantage Elan). The following sequences were performed: sagittal plane T2W, sagittal plane T1W, sagittal plane short-tau inversion recovery (STIR), dorsal plane STIR, transverse plane T2W, transverse plane T1W, transverse plane T2* GRE, sagittal plane T1W post-contrast, transverse plane T1W post-contrast, and sagittal plane 3D T1W magnetization prepared—rapid gradient echo (MPRAGE). MRI revealed a large, moderately well-defined and slightly lobulated space-occupying lesion, extending from the mid-body of L5 to the caudal aspect of L6 (Figures 1B–G). The lesion originated from the right side and occupied or compressed most of the spinal cord parenchyma. At the level of L6-7, it appeared to extend and affect the right ventral nerve root (Figure 1H). The caudal aspect of the lesion was ill-defined and may cause mass effect on the cauda equine nerves. The lesion was slightly heterogeneous, mainly hyperintense on T2-weighted (T2W) and STIR when compared to the spinal cord, mildly hypointense on T1W images, and had mild to moderate slightly heterogeneous contrast enhancement. Small areas of hypointensity/signal void within the lesion were appreciated from the T2* sequence. No golf tee sign or dural tail sign was conclusively observed. The large volume of the mass prohibited conclusions on the exact localization of the mass lesion (i.e., intradural, intradural/extramedullary, or extradural) but an intradural and possibly at least partially intramedullary localization was suspected. Despite the size of the mass, no surrounding spinal cord oedema or dilation of the central canal were noted.

**Figure 2:**
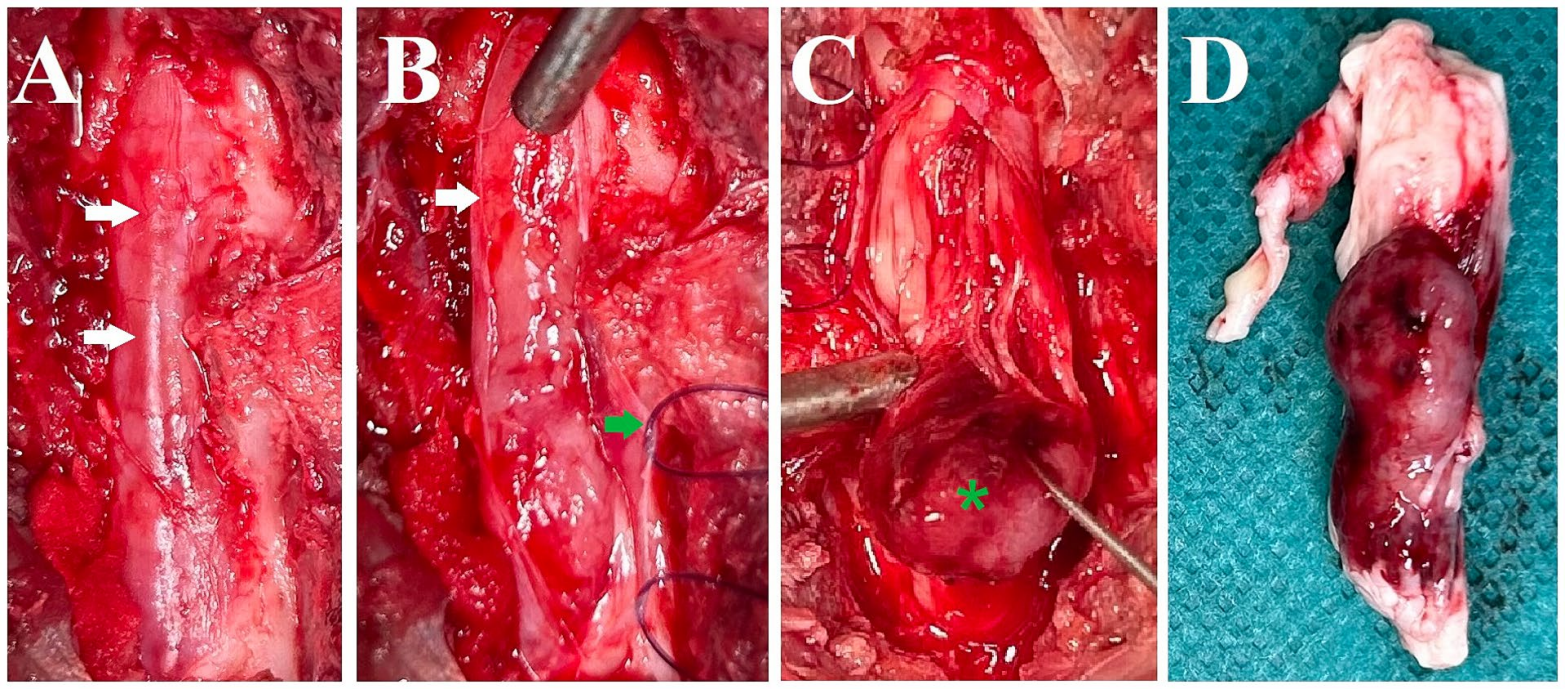
Intraoperative pictures and a picture after removal of the spinal cord segments and nerve roots with the mass. **(A)** Dorsal aspect of the spinal canal after dorsal laminectomy. The dura mater is intact and the mass lesion is seen as red discoloration through it (white arrows). **(B)** Dura mater reflected (white arrow: edge of cut dura mater, green arrow: dura mater with stay suture). **(C)** Blunt dissection and caudal reflection of the main bulk of the mass lesion (green asterisk). It was attempted to bluntly dissect the mass but it was found to be adherent to or fused with the spinal cord parenchyma and spinal nerve roots. **(D)** Lateral (right) view of the removed spinal cord segments, nerve roots, and associated mass (directly post-mortem).

Treatment was initiated with prednisolone (0.6 mg/kg twice daily per os) but over the course of five days, the cat’s condition deteriorated to the extent that it could barely ambulate and exhibited urinary and fecal incontinence. Due to this swift deterioration, surgery was elected after discussion with the owner. A dorsal laminectomy was performed at L5-L6. The intradural and predominantly extraparenchymal red mass was visible through the dura mater (Figure 2A). After reflecting the dura mater (Figure 2B), using a surgical microscope, it was attempted to bluntly dissect the mass but it was found to be adherent to or fused with the spinal cord parenchyma and spinal nerve roots (Figure 2C). Following intraoperative consultation with the owner, including the options of further attempting to remove the tumor versus euthanasia as the owner had indicated the wish to be informed about possibly negative prognostic findings including difficulty removing the tumor, euthanasia was elected. The mass, along with the affected spinal cord segments and nerve roots, was extracted (Figure 2D), fixed in 10% buffered formalin, and submitted for histology.

### Pathology

**Figure 3:**
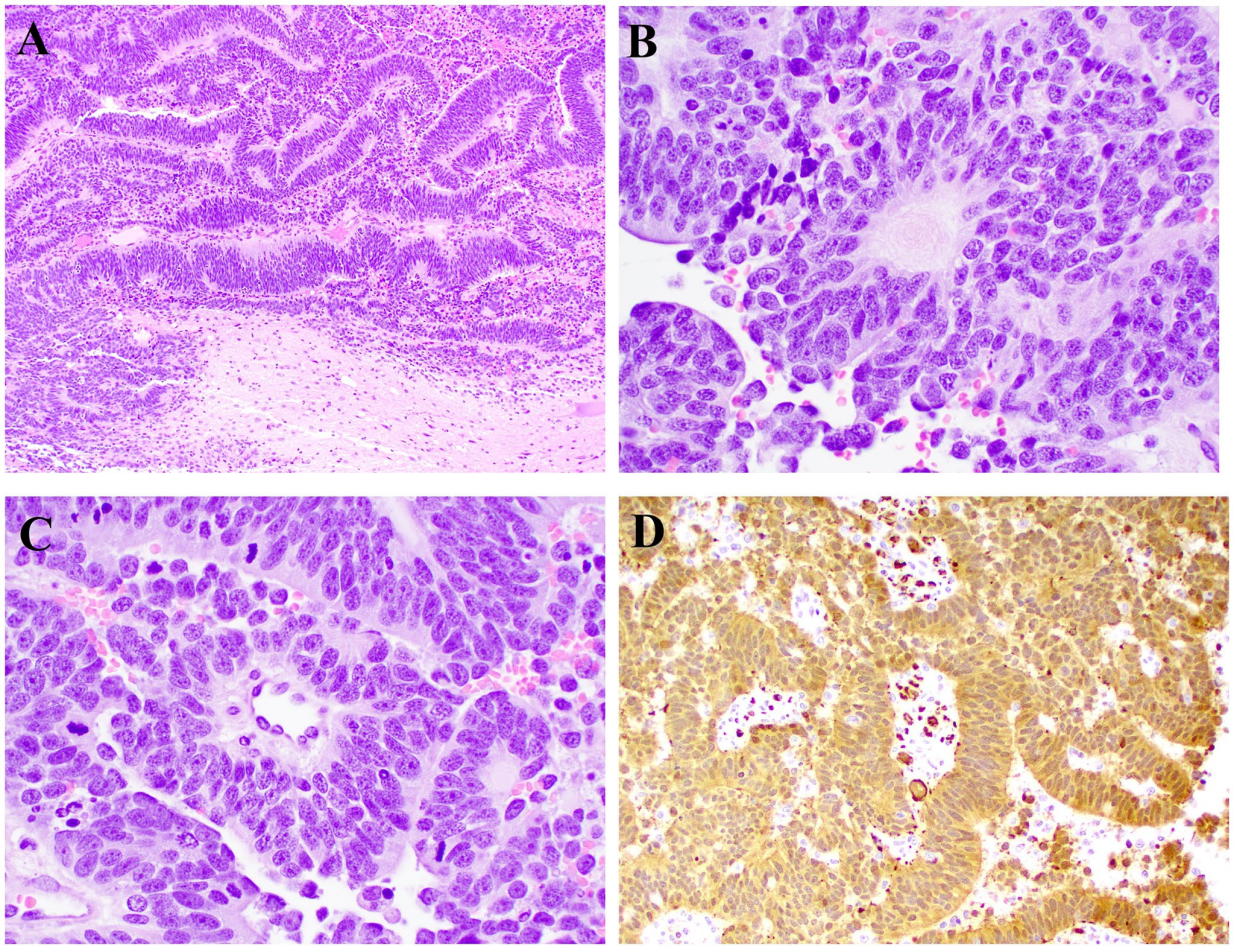
Microscopic photographs of histology. **(A)** Neoplastic cells arranged in rowing and pseudostratified patterns aligned perpendicularly to the supporting fibrovascular stroma. HE, 10X. **(B)** Neoplastic cells palisade around a central core resembling the neuroparenchyma (neuroblastic rosette). HE, 40X. **(C)** Neoplastic cells palisade around a central blood vessel (pseudorosette). HE, 40X. **(D)** Neoplastic cells have widespread cytoplasmic immunolabeling for PGP9.5. IHC, 20X.

The neoplasm and attached structures were sectioned, processed routinely for histology, and stained with hematoxylin and eosin. Histologically the neoplasm was poorly demarcated and primarily extraparenchymal but had areas of infiltration into the white matter of the spinal cord and adjacent nerve roots. Neoplastic cells were arranged in rowing and pseudostratified patterns aligned perpendicularly to the supporting fibrovascular stroma (Figure 3A). Neoplastic cells had a tall and columnar, eosinophilic cytoplasm with indistinct borders and round to oval nuclei with finely stippled to coarse chromatin and 1–2 nucleoli. Neoplastic palisading around a central core resembling the neuroparenchyma (neuroblastic rosettes, Figure 3B) or around blood capillaries (pseudorosettes, Figure 3C) were distributed throughout the neoplasm. The mitotic count was 73 in 2.37 mm^2^ (10 FN22/40X fields). Anisocytosis and anisokaryosis were moderate.

IHC was independently evaluated by two pathologists from different institutions. Sections of the neoplasm were subjected to IHC for pancytokeratin, oligodendrocyte transcription factor 2 (OLIG2), glial fibrillary acidic protein (GFAP), neuron nuclei protein (NeuN), neuron specific enolase (NSE), neurofilament (NF), synaptophysin (SYN), and protein gene product 9.5 (PGP9.5). Feline spinal cord (OLIG2, GFAP, NeuN, NSE, NF, SYN, and PGP9.5) and haired skin (pancytokeratin) were used as external control tissues. In addition, spinal cord adjacent to the neoplasm was used as internal control for all immunomarkers except pancytokeratin. There was robust and widespread cytoplasmic PGP9.5 immunolabeling throughout the neoplasm (Figure 3D). All other IHCs were negative. Immunolabeling in the external and internal controls was adequate for all IHCs. Histologic findings were presumed to be consistent with a final diagnosis of an embryonal neoplasm affecting the spinal cord and adjacent nerves, including those of the cauda equina.

## Discussion

Primary spinal cord tumors are infrequently reported in cats, accounting for approximately 16% of all spinal cord and vertebral neoplasms (1). Spinal embryonal tumors (SETs) are exceedingly rare, with just a few case reports in the veterinary literature (17, 23, 24). In 2024, a case report was published describing a spinal cord medulloepithelioma (MEPL) in a 14-month-old cat, also localized at L5-6. Both cases involved an embryonal tumor at a comparable spinal level and the clinical presentation was comparable, although our patient displayed asymmetrical rather than symmetrical deficits and the MEPL case was non-ambulatory. The MEPL was surgically removed, although the authors reported difficulty to distinguish tumor tissue from edematous spinal cord parenchyma. Indeed, the intraoperative photographs bear resemblance to that of our case. The cat initially recovered but tumor regrowth was reported at 14 months (17). To the best of our knowledge this is the second SET at the caudal lumbar vertebral level in a feline patient and should be included specifically in the differential diagnosis of cats presenting with lumbosacral / sacrocaudal myelopathy, conus medullaris syndrome, or cauda equina syndrome. However, the currently limited number of reported cases does not discount the possibility of this type of tumor affecting more cranial spinal cord segments.

In our case, based on the localization in the spinal canal on MRI and post-mortem examination, the mass primarily affected the L7-Cd spinal cord segments and associated nerve roots (25). Lesions at the L4-S3 and caudal segments commonly affect both spinal cord segments and nerve roots that makes it difficult to distinguish them from lesions affecting the conus medullaris, nerve roots, or spinal nerves of the cauda equina at a more caudal level in the spinal canal. Subtle neurological differences can distinguish them. The conus medullaris includes the sacral and caudal segments (S1-Cd5), while the cauda equina consists of paired nerve roots extending lateral and caudal to the conus medullaris, exiting through lumbar, sacral, and caudal foramina. Both conus medullaris syndrome (CMS) and cauda equina syndrome (CES) cause urinary, anal, and rectal dysfunction, tail and saddle anesthesia, and lower motor neuron paraparesis or tail paralysis (26). However, clinically they can be differentiated as follows: CES may present unilaterally, while CMS is always bilateral (26). Reported sensory differences between CES and CMS are largely inferred from human literature (27). Veterinary descriptions of CMS are scarce and clinical determination of sensory dysfunction is subjective making clinical differentiation between CMS and CES may be and remain difficult. In this case, neurological deficits were more pronounced on the right side, though both sides were affected. Specific sensory regions were not tested.

In humans SETs are rare, aggressive, often metastatic at diagnosis, and are associated with poor prognosis (28, 29). SETs are most commonly located in the thoracic and lumbar regions, with sacral tumors being rarely reported (30). Primary or secondary nervous system tumors located at the cauda equina are rare, comprising 3.5% of all spinal cord tumors (31). Treatment consist of surgical reduction or resection, chemotherapy, and radiation. There is not enough data to draw information on the clinical behavior of these embryonal neoplasms in cats or other domestic animals. Nevertheless, surgical removal seems unlikely to be curative due to invasiveness of the neoplasm in the two reported feline cases so far [ours and that reported by Aytaş et al. (17)].

MRI features of SET in people are variable, but commonly reported imaging characteristics are restrictive diffusion, mild to moderate contrast enhancement, and lack of perilesional oedema (32, 33). In the case reported here, diffusion weighted imaging was not available, but the contrast enhancement pattern and the lack of surrounding edema can be regarded as similar to MRI characteristics of SET as reported in people. However, these findings are not unusual in other spinal neoplasms reported in cats, such as gliomas or lymphomas. The contrast enhancement pattern in gliomas can vary from none to marked and are located intramedullary (intraparenchymal) (2, 4). The SET in the cat described here was too large to distinguish the exact location; however, an intradural location was highly suspected, together with spinal nerve root involvement. The previously reported MEPL case also shared similar imaging features, appearing hyperintense on T2W images and exhibiting contrast enhancement. In the MEPL case, the lesion was deemed to be intradural but extramedullary based on MRI but was adhered to and likely invading spinal cord parenchyma based on intraoperative findings (17). Spinal lymphoma can have extramedullary and intramedullary localizations, with possible extension into vertebrae and adjacent soft tissues. However, with regard to the spinal cord, they tend to be focal and extramedullary with variable contrast enhancement (34). Therefore, its MRI features are non-specific and may overlap with those described for the two feline cases with SET, that can be regarded as non-specific as well.

Although neoplastic cells had cytoplasmic immunolabeling for PGP9.5 in our case, no immunolabelling was observed for conventional neuronal immunomarkers such as NeuN, NSE, NF, and SYN, or glial immunomarkers (GFAP and OLIG2). Immunohistochemistry in the previously reported feline MEPL case revealed no immunolabelling for GFAP, NeuN, SYN, PCK and OLIG2, but showed positive labeling for NSE and VIM, consistent with reports of human MEPLs (17). Although embryonal neoplasms are reportedly positive for PGP9.5, this is not a confirmatory immunomarker for these tumors, and labeling should always be interpreted in the context of neoplastic cell morphology and other IHC tests (19, 35). Although our neoplasm had morphologic features that support an embryonal origin and we presumed the tumor to be a SET, the IHC findings cannot be regarded as irrefutable confirmation. Nevertheless, the extensive IHC together with the HE histological findings justify the final diagnosis of an embryonal tumor.

In conclusion, we report the second case of a SET in a cat, localized in the caudal lumbar spinal canal. Including this differential diagnosis in cats, especially of fairly young age (reported cases included a 14-month-old cat and our 5-year-old cat), seems prudent.

The authors would like to thank IVC Evidensia via the Group Veterinary Medical Board for providing funds for the publication of this article.

## Data Availability

The original contributions presented in the study are included in the article/Supplementary material, further inquiries can be directed to the corresponding authors.

## Supplementary material

SUPPLEMENTARY VIDEO 1 Clinical presentation of the cat of this report. The cat exhibits a moderate ambulatory paraparesis and mild proprioceptive ataxia with plantigrade stance and a flaccid tail paresis.
